# Supplementary material for: Transcriptional Silencing of 35S rDNA in Tragopogon porrifolius Correlates with Cytosine Methylation in Sequence-Specific Manner
Source: Int J Mol Sci. 2024 Jul 9;25(14):7540. doi: 10.3390/ijms25147540 (PMC11276851; doi:10.3390/ijms25147540)
Supplement: Supplementary file 1 [file ijms-25-07540-s001.zip › Supplementary Materials 1_Figuress A1-A10; Tables A1, A2, A4..A8.pdf]

Transcriptional silencing of 35S rDNA in *Tragopogon porrifolius* correlates differentially with cytosine methylation in distinct sequence contexts

Roman Matyášek, Radka Kalfusová, Alena Kuderová, Kateřina Řehůřková, Jana Sochorová and Aleš Kovařík

Supplementary Material (Tables S1, S2, S4, S5, S6, S7, S8 and S9 ; Figures S1 – S10)

Table S1. Pairwise sequence homology between promoter-like sequences

P/P	P_1	P_2-S	P_2-L	P_3	P_4-S_b	P_4-L_d	P_4-L_c	P_4-S_a	P_5-L	P_5-S	P_6-L	P_6-S	P_7	down-mean
P_1		51.9	51.9	49.4	48.1	48.1	50.6	50.6	52.6	54.3	49.3	49.3	49.4	50.46
P_2-S	97.2		98.6	74.7	53.9	53.9	53	54.2	64.6	64.6	73.3	74.7	75.7	66.09
P_2-L	97.2	100		76	52.6	52.6	51.8	53	63.4	63.4	74.7	76	77	65.92
P_3	95.8	95.8	95.8		50.6	50.6	54.5	55.8	79.7	78.5	**98.6**	**97.2**	**93.1**	71.56
P_4-S_b	97.2	97.2	97.2	98.6		100	68.4	67.1	50	50	50.6	50.6	53.8	57.98
P_4-L_d	95.8	95.8	95.8	97.2	98.6		68.4	67.1	50	50	50.6	50.6	53.8	57.98
P_4-L_c	95.8	95.8	95.8	97.2	98.6	97.2		98.6	50.6	48.2	53.2	55.1	56.4	59.07
p_4-S_a	97.2	97.2	97.2	98.6	100	98.6	98.6		50.6	48.2	54.5	56.4	56.4	59.38
P_5-L	95.8	95.8	95.8	97.2	98.6	97.2	100	98.6		98.6	78.5	78.8	75.9	66.11
P_5-S	95.8	95.8	95.8	97.2	98.6	97.2	97.2	98.6	97.2		77.2	77.5	74.7	65.43
P_6-L	95.8	95.8	95.8	**100**	98.6	97.2	97.2	98.6	97.2	97.2		98.6	**91.7**	70.90
P_6-S	95.8	95.8	95.8	**100**	98.6	97.2	97.2	98.6	97.2	97.2	100		**90.3**	71.26
P_7	97.2	97.2	97.2	98.6	100	98.6	98.6	100	98.6	98.6	98.6	98.6		70.68
up-mean	96.38	96.62	96.62	97.67	98.48	97.20	97.43	98.48	97.43	97.20	97.67	97.67	98.48	

Core promoters (72 bp) located upstream of TIS are compared bellow the mean diagonal. Corresponding 72 bp regions located downstream of TIS are compared above the diagonal. Mean for each region is highlighted by gray background. Individual variants of P_2, P_4, P_5 and P_6 are associated with respective rDNA variants (Figure S2). The highest similarity, detected between P_3 and P_6, are underlined in bold.

Table S2. Copy numbers of individual rDNA variants in por1 and por2 genomes

a) Total copy numbers of rDNA in por1 and por2

sample	2C genome size[(1)]		18SrDNA (1815 bp)		
	[Mb]	total reads	reads	GP[(2)] [Mbp]	CN[(3)]
por1-34_leaf	6453.3	393997426	579592	9.4932	5230
por1-34_root tips	6453.3	394807698	604186	9.8757	5441
por1_seedlings	6453.3	396603644	531917	8.6550	4769
por1_total	6453.3	1185408768	1715695	9.3401	5146
por2-15_leaf	6453.3	394325336	1298177	21.2452	11705
por2-15_root tips	6453.3	395443216	1587149	25.9009	14270
por2-134_leaf	6453.3	345564026	1134858	21.1931	11677
por2-134_root tips	6453.3	348372212	985180	18.2496	10055
por2_seedlings	6453.3	351342680	1033284	18.9789	10457
por2_total	6453.3	1835047470	6038648	21.2361	11700

(1) The same value of 2C genome size (https://cvalues.science.kew.org/) was used for all *T. porrifolius samples*.

(2) Genome proportion (GP) of 18S rDNA was calculated according to equation:

$$GP\ [\boldsymbol{Mbp}] = \frac{(mapped\ 18S\ rDNA\ reads)}{(total\ reads)} * 2C$$

(3) The rDNA copy number (CN) was calculated according to equation:

$$CN = \frac{1000000}{1850} * GP$$

b) Copy numbers of individual rDNA variants in por2 estimated from Illumina and PacBio sequencing

	number of reads[(1)]					ratios between variants[(2)]			copy numbers[(3)]			proportion [%]		
	L	S	L^a	L^s	S+L^s	L/S	(S+L^s)/L^a	L^a/L^s	S	L^a	L^s	S	L^a	L^s
Illumina	49728	25998	11628	nd	15915	1.9128	1.3687	1.8003	4016	4939	2744	34.33	42.22	23.45
PacBio	60	30	42	18	48	2.0000	1.1429	2.3333	3900	5460	2340	33.33	46.67	20.00

(1) For Illumina reads, S- and L- rDNA variants were distinguished in ETS2 regions I-IV, while (S + L^s)- and L^a-rDNAs were distinguished in ETS2 regions V – VI (Figure S5a).

(2) For Illumina reads, the ratio L^a/L^s was computed according to equation:

$$L^a/L^s = (A+1)/(A*B - 1)$$

where A = L/S and B = (S + L^s)/L^a

(3) Computed from total copy number shown in the previousTable

Table S4. List of primers

primer	5'-3' sequence	orientation	position in rDNA	experiment
ETS2F	CGAAAGTTGGACAACGACGA	forward	ETS2; Figure 2c	CAPS; Figure 2c
ETS2R	CGATACATGGACGACGGTCA	revers	ETS2; Figure 2c	
NTSF	AAATCCGAGCTCGTTTGTGGGAG	forward	NTS; Figure 1b	PCR Figure 1c; IGS probe
ETS2R1	CCAACATCGCCCTCCACGCTTCTTC	revers	ETS2; Figure 1b	
ETS2F88	AGTGTTGATTGGTCCCTTGGA	forward	ETS2; Figure 2d	CAPS; Figure 2d
ETS2R88	AGCTAAATCACTCGATAAGGGCA	revers	ETS2; Figure 2d	

Table S5. **Statistics of Bisulfite - Illumina sequencing**

Sample	bases[1]	reads[2]	GC[3]	AT[4]	Q20[5]	Q30[6]
por1-34-leaf	59,493,611,326	393,997,426	26.16	73.84	97	91.63
por1-34-root tips	59,615,962,398	394,807,698	25.84	74.16	97.1	91.75
por1-seedlings	59,887,150,244	396,603,644	26.75	73.25	97.2	92.13
por2-134-leaf	52,180,167,926	345,564,026	25.7	74.3	96.8	91.24
por2-134-root tips	52,604,204,012	348,372,212	24.93	75.07	96.9	91.41
por2-seedlings	53,052,744,680	351,342,680	25.94	74.06	97	91.53
por2-15-leaf	59,543,125,736	394,325,336	25.37	74.63	97.1	91.84
por2-15-root tips	59,711,925,616	395,443,216	26.26	73.74	97.08	91.82

[1] Total number of bases sequenced.
[2] Total number of reads. For Illumina paired-end sequencing, this value refers to the sum of read1 and read 2
[3] GC content in percentages.
[4] AT content in percentages.
[5] Percentages of bases that have phred quality score of over 20.
[6] Percentages of bases that have phred quality score of over 30.

Table S6. List of biosamples with deposited NGS data implemented in BioProject ID PRJNA634996

experiment	Biosample accession	name	description
PacBio-Seq	SAMN16261961	por1_leaf	DNA from por1 leaves
	SAMN15014845	por2_leaf	DNA from por2 leaves
RNA-Seq	SAMN26145753	Por2RNA_134leaf	RNA from por2 leaves
	SAMN26145754	Por2RNA_134root	RNA from por2 root tips
Bisulfite-Seq	SAMN25610388	Por1_34leaf	bisulfite treated DNA from por1-34 leaves
	SAMN25610389	Por1_34root	bisulfite treated DNA from por1-34 root tips
	SAMN25610390	Por1_seedlings	bisulfite treated DNA from por1 seedlings
	SAMN25610391	Por2_134leaf	bisulfite treated DNA from por2-134 leaves
	SAMN25610392	Por2_134root	bisulfite treated DNA from por2-134 root tips

SAMN25610393	Por2_seedlings	bisulfite treated DNA from por2
SAMN29021381	Por2_15leaf	bisulfite treated DNA from por2-15 leaves
SAMN29021382	Por2_15root	bisulfite treated DNA from por2-15 root tips

Table S7. Relative contents of ETS2 and 18S rRNA transcripts in indicated tissues (Source data for Figure 2a)

a) Number of total and mapped Illumina reads from RNA-seq

Tissue[(1)]	total reads			18S rRNA[(2)]			ETS2[(2)]		
	por1	por2-1	por2-2	por1	por2-1	por2-2	por1	por2-1	por2-2
leaf	105285864	106921822	123603460	8034944	7715914	10988574	12597	13891	31378
root tips	54539110	109495178	131959862	5894109	11521553	13956032	22627	67677	87200
seedlings	196755736	226899202		20307938	23380144		36866	32106	

(1) Leaf and root tips were analyzed in one por1 and two por2 (por2-1 and por2-2) individuals, whereas one sample of seedlings was analyzed for each por1 and por2 lineage

(2) Illumina reads were mapped to the equally long (600 bp) regions of each 18S rRNA and ETS2.

b) Normalized contents of ETS2 and 18S rRNA

tissue	10000 x ETS2/total RNA					1000 x ETS2/18S rRNA					50x18S rRNA/total RNA				
	por1	por2-1	por2-2	mean	SD	por1	por2-1	por2-2	mean	SD	por1	por2-1	por2-2	mean	SD
leaf	1.1965	1.2992	2.5386	1.6781	0.5737	1.5678	1.8003	2.8555	2.0745	0.5207	3.8158	3.6082	4.4451	3.9564	0.3258
root tips	4.1488	6.1808	6.6081	5.6459	0.9981	3.8389	5.8739	6.2482	5.3204	0.9876	5.4036	5.2612	5.288	5.3176	0.0573
seedlings	1.8737	1.415		1.6443	0.2294	1.8153	1.3732		1.5943	0.2211	5.1607	5.1521		5.1564	0.0043

c) Comparative statistics

sample		ETS2/total RNA		ETS2/18S rRNA		18S rRNA/total RNA	
1st	2nd	F-test [*p2*]	t-test [*p2*]	F-test [*p2*]	t-test [*p2*]	F-test [*p2*]	t-test [*p2*]
leaf	root tips	0.48848	0.01044	0.43771	0.01858	0.058504412	0.005963521
leaf	seedlings	0.58701	0.95745	0.61322	0.43735	0.019729894	0.037818346
root tips	seedlings	0.34388	0.02756	0.33616	0.03199	0.113480059	0.064893272

Number of reads mapped to ETS2 (600 bp) were related to number of reads mapped to 18S rRNA region of the same length.

Table S8. Proportions of individual S-rDNA variants in por1 DNA and primary transcripts

rDNA	proportion in genome [%]				proportion in transcript [%]				comparisons between transcript and genome(1)					
									F-test	t-test	transcriptome/genome			
	leaf	roots	seedl.	mean	leaf	roots	seedl.	mean	*p2*	*p2*	leaf	roots	seedl.	mean
S_1	72.15	73.88	78.12	74.72	67.79	71.64	67.91	69.11	0.67277	0.06180	0.940	0.970	0.869	0.926
S_2	11.62	12.79	11.86	12.09	20.56	16.70	20.13	19.13	0.15917	0.00524	1.770	1.305	1.698	1.591
S_3	9.35	5.71	3.45	6.17	8.43	11.37	10.81	10.20	0.43192	0.10608	0.902	1.991	3.131	2.008
S_4	3.33	3.85	3.07	3.42	3.15	0.22	1.12	1.50	0.13316	0.09939	0.947	0.058	0.366	0.457
S_5	3.56	3.76	3.50	3.61	0.08	0.08	0.02	0.06	0.11573	0.00000	0.021	0.020	0.005	0.015
$S_{1,3,4}$	84.82	83.45	84.64	84.30	79.37	83.23	79.85	80.82	0.22487	0.05368	0.936	0.997	0.943	0.959
$S_{1,4,5}$	79.04	81.50	84.69	81.74	71.02	71.94	69.05	70.67	0.42552	0.00387	0.899	0.883	0.815	0.866
$S_{2,3}$	20.96	18.50	15.31	18.26	28.98	28.06	30.95	29.33	0.42552	0.00387	1.383	1.517	2.021	1.640

(1) Students t- test was used to statistical evaluations of differences between proportions of individual rDNA variants in genomes and transcriptomes. Ratios between proportions in transcriptome and genome was used to evaluate transcriptional efficiency of individual variants - source data for Figures 2d and S9c, e.

Table S9. Mapped Bisulfite-modified Illumina reads to individual ETS2 regions and characteristics of individual DNA strands (plus and minus)

region	G+C	G/C	no of CHHs		sample	mapped and trimmed reads		
	[%]	plus	plus	minus		plus	minus	plus/minus
I	50.00	2.40	3	30	por1-34_leaf	21948	1098	19.99
					por1-34_root tips	25015	1118	22.37
					por1_seedlings	21963	1033	21.26
					por2-134_leaf	47922	1917	25.00
					por2-134_root tips	43934	1841	23.86
					por2-15_leaf	54568	2676	20.39
					por2-15_root tips	66853	309	216.35
					por2_seedlings	41072	2197	18.69
II	58.33	1.59	14	27	por1-34_leaf	6031	515	11.71
					por1-34_root tips	4913	476	10.32
					por1_seedlings	4234	524	8.08
					por2-134_leaf	7619	586	13.00
					por2-134_root tips	7222	449	16.08
					por2-15_leaf	11515	1280	9.00
					por2-15_root tips	12888	1275	10.11
					por2_seedlings	7676	899	8.54
III	46.96	1.57	16	26	por1-34_leaf	5413	4517	1.20

					por1-34_root tips	4728	4911	0.96
					por1_seedlings	3863	4464	0.87
					por2-134_leaf	5709	6904	0.83
					por2-134_root tips	6105	6431	0.95
					por2-15_leaf	10168	10429	0.97
					por2-15_root tips	11218	12779	0.88
					por2_seedlings	6309	6950	0.91
IV	44.70	1.19	19	21	por1-34_leaf	4519	5813	0.78
					por1-34_root tips	3797	6164	0.62
					por1_seedlings	3166	5757	0.55
					por2-134_leaf	3913	8948	0.44
					por2-134_root tips	4357	7665	0.57
					por2-15_leaf	10371	13892	0.75
					por2-15_root tips	11114	16265	0.68
					por2_seedlings	5084	8621	0.59
V	49.58	1.19	22	24	por1-34_leaf	5951	5194	1.15
					por1-34_root tips	5200	4680	1.11
					por1_seedlings	4321	4020	1.07
					por2-134_leaf	4913	7287	0.67
					por2-134_root tips	5303	6339	0.84
					por2-15_leaf	11858	11640	1.02
					por2-15_root tips	13185	13080	1.01
					por2_seedlings	5801	6997	0.83
VI	44.29	1.38	16	26	por1-34_leaf	4943	4524	1.09
					por1-34_root tips	4032	4539	0.89
					por1_seedlings	3798	4213	0.89
					por2-134_leaf	7657	5915	1.29
					por2-134_root tips	7090	5541	1.28
					por2-15_leaf	8298	10510	0.79
					por2-15_root tips	10102	11799	0.86
					por2_seedlings	7463	6187	1.21

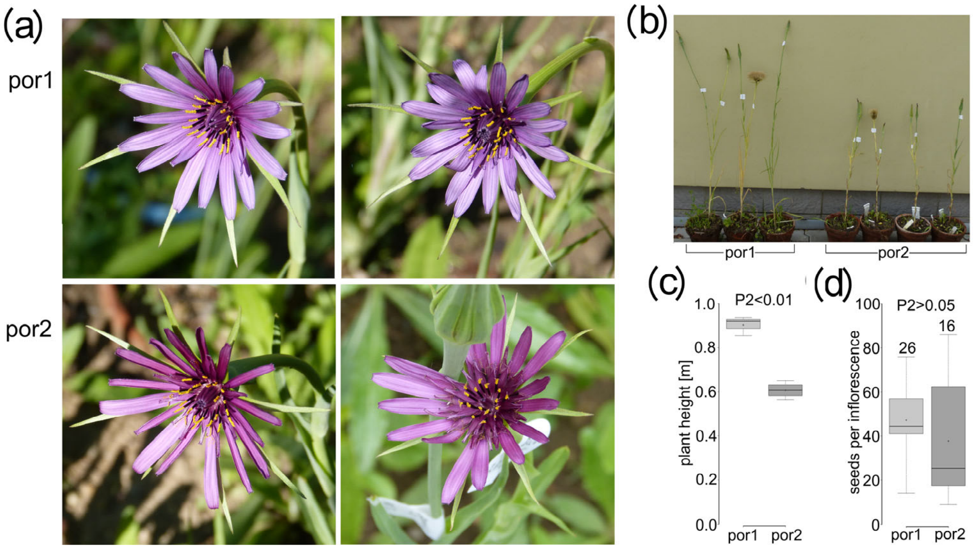

Figure S1. Phenotype characterization of por1 and por2 lineages. (a) Flower morphology is demonstrated on two plants of each por1 and por2 variant. Relative lengths of ligules and bracts suggest that both variants belong to *T. porrifolius* subsp. porrifolius. **(b)** Mature plants after flowering period. **(c)** The por1 mature plants are significantly taller than por2 ones. **(d)** Average number of seeds per inflorescence was insignificantly higher for por1 lineage. Numbers of analysed inflorescences are shown above box plots. Box plots are constructed of minimum, 1st quartile, median, 3rd quartile, maximum and mean (+).

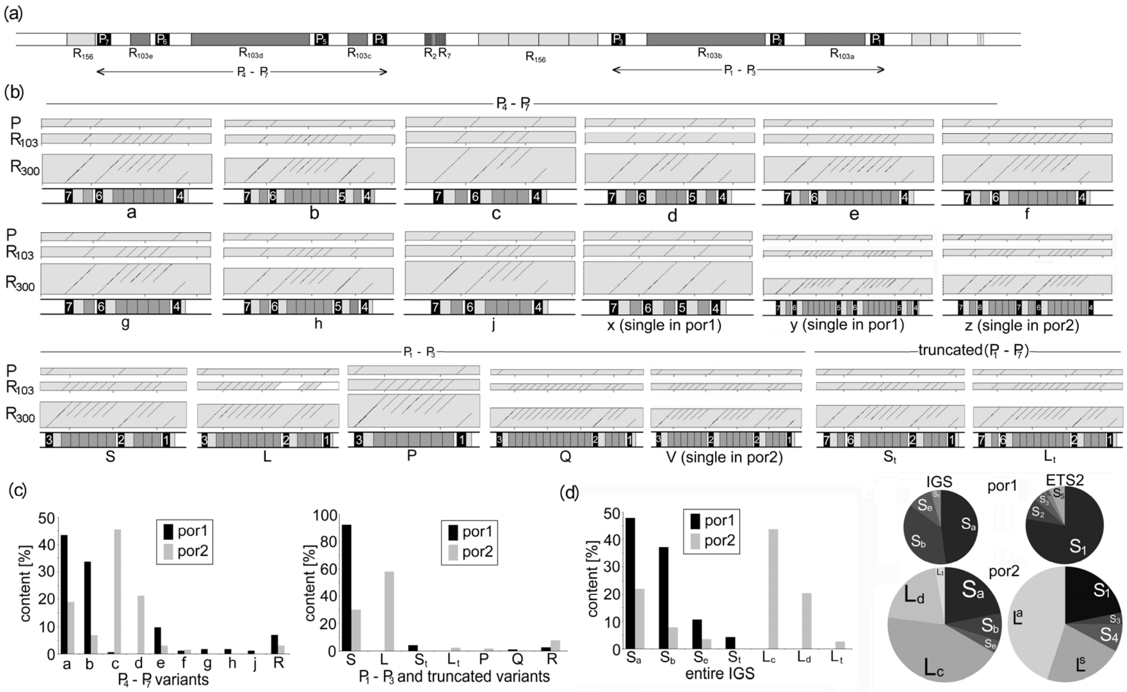

Figure S2. Fine structural variations within each L- and S-rDNA in por1 and por2. (a) Highly variable but considerably long P - R_{103} repeat arrays were split into two regions covering P_1 - P_3 and P_4 - P_7 promoters, whose variability were analysed separately. **(b)** Twelve

(a, b, c, d, e, f, g, h, j, x, y and z) and five (S, L, P, Q and V) arrangements of P and R_{103} repeats were detected in P_4-P_7 and P_1-P_3 regions, respectively, using two types of dot-plots constructed for: (i) ~80 bp long core promoter P (black rectangles numbered according to Figure 1B), and (ii) 103-104 bp long R_{103} repeats (dark gray). It is necessary to emphasize that although x-arrangement was detected only once in por1, it can sometimes be broadly amplified as occurred in some lineages of allopoplyploid *T. mirus*. Rarely occurring simultaneous deletion of R_2, R_7 and R_{156} repeats resulted in truncated rDNA variants S_t and L_t (P_1 – P_7). The scale on the x-axis is 500 bp. **(c)** Proportions of individual rDNA variants in por1 and por2 were estimated from PacBio sequencing separately for both P_4-P_7 and P_1-P_3 regions. Very rarely occurring variants are considered together as R. **(d)** Only a-S (S_a), b-S (S_b), e-S (S_e), c-L (L_c) and d-L (L_d) connections between variants from P_4-P_7 and P_1-P_3 regions were confirmed by sufficiently long PacBio reads and proportions of entire abundant rDNA variants (S_a, S_b, S_e, L_c and L_d were inferred from both regions and expressed using a bar and a pie chart, the latter of which can also express the relative copy numbers of rDNA in por1 and por2 (Table S2). Proportions of individual rDNA variants were estimated from variability in both IGS and ETS2.

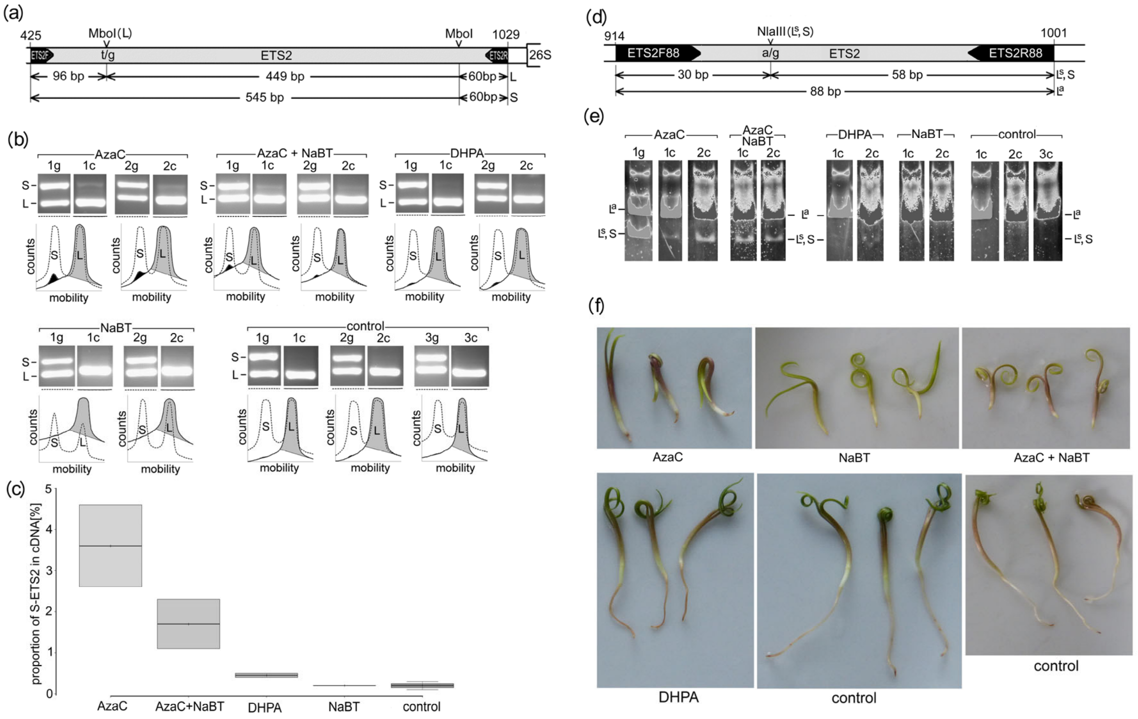

Figure S3. Partial activation of silent rDNA by cytosine - hypomethylating drugs. (a) Targets for pair of primers ETS2F-ETS2R and respective MboI that differentiates ETS2 between S- and L-rDNA variants are related to genic TIS. **(b)** Genomic DNAs (g) or cDNAs (c) from two or three (control) por2 plants, treated with AzaC, DHPA and NaBT (Material and methods), were subjected to corresponding CAPS analyses. Restricted DNA fragments were separated in 2% agarose, stained with EtBr and relative content of individual fragments were

evaluated densitometrically and expressed as total counts. Dotted and solid curves correspond to gDNA and cDNA, respectively. Activated S-rDNA in cDNA is highlighted in black **(c)** Relative abundances of activated S-rDNA variants were expressed as box plots built of maximum, median and minimum**. (d)** Targets for pair of primers ETS2F88-ETS2R88 and respective NlaIII that differentiates between silent (S + L^s) and active (L^a) rDNA are related to genic TIS. **(e)** Genomic DNA (g) or cDNA (c) of two or three (control) plants, treated with indicated drugs, were subjected to corresponding CAPS analyses. Restricted DNA fragments were separated in 10% polyacrylamide. **(f)** Phenotypes of individual seedlings just before analyses. Both AzaC and NaBT predominantly affected length of roots whereas DHPA treatments resulted in shorter plants as entire.

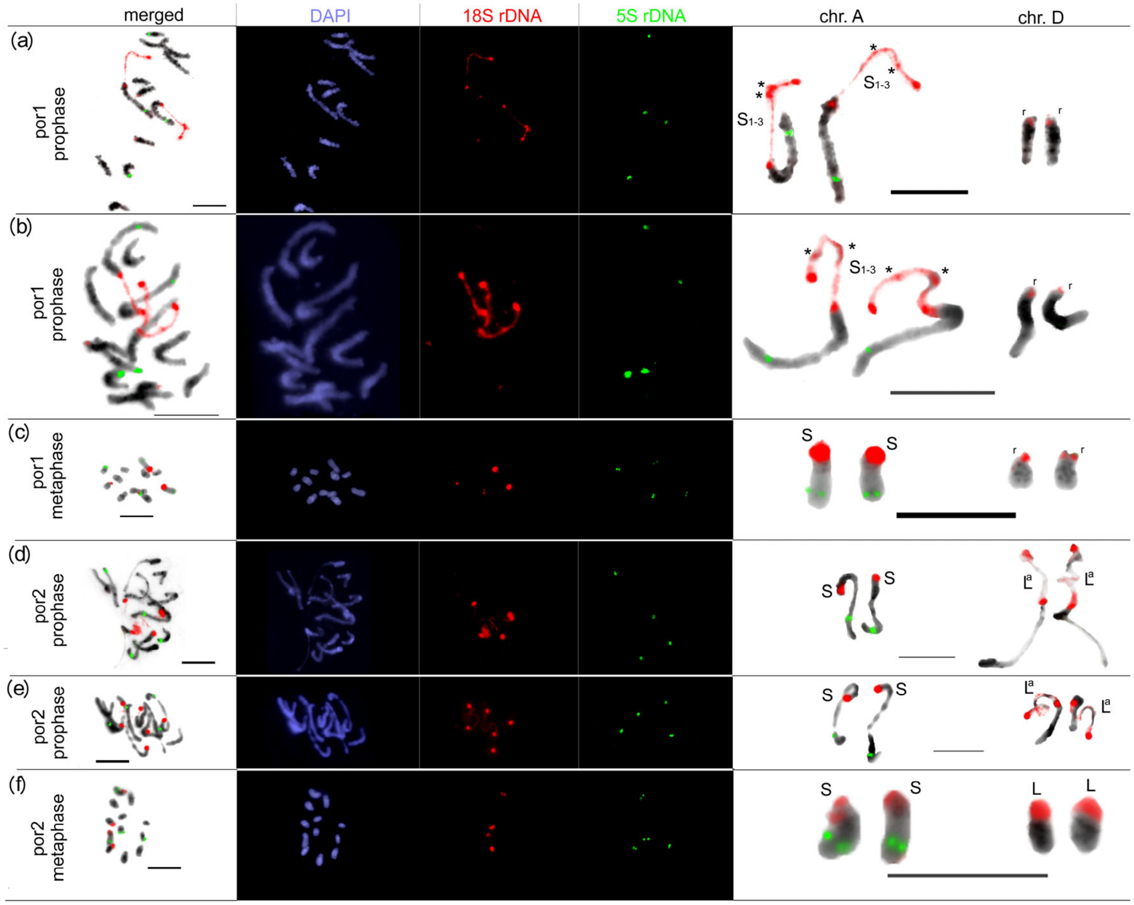

Figure S4. Chromosomal organizations of 35S rDNA greatly differ between por1 and por2. Fluorescence in situ hybridizations of 18S rDNA (red) and 5S rDNA (green) to prophase (**a, b, d** and **e**) and metaphase (**c** and **f**) chromosomes in por1 (**a, b** and **c**) and por2 (**d, e** and **f**). Presumptive mutual localizations of individual rDNA variants are depicted on excised and enlarged chromosomes A and D on the right side. In por1, the decondensed 35S rDNA region on each chromosome A is split by two condensed foci (asterisk) into three decondensed subregions which may correspond to the three transcribed rDNA variants $S_{1\text{-}3}$ (Figure 2e). In contrast, the decondensed 35S rDNA regions on each chromosome D is uninterrupted and perhaps correspond to single transcribed rDNA variant L^a, detected in por2. Chromosomes were counterstained with DAPI (blue). Representatives were selected from 34 analyzed metaphases (prophases) for each por1 and por2.

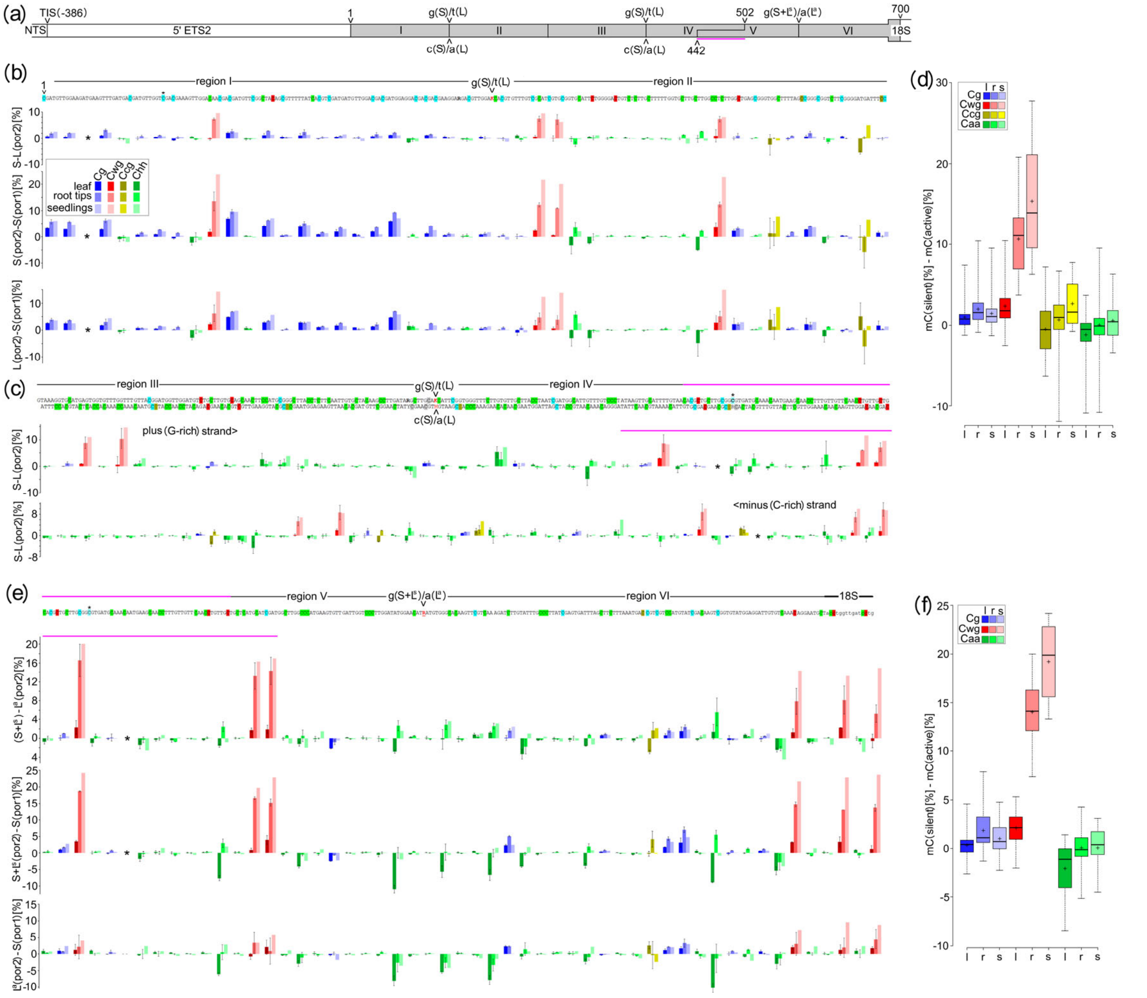

Figure S5. Differences between methylation of each respective cytosine in indicated pairs of rDNA variants. **(a)** Six regions (I – VI) in ETS2 were delimited by (i) two G>T(C>A) abundant transversions, distinguishing bisulfite - modified ETS2 between S-rDNA and L-rDNA, and (ii) one G>A(C>T) abundant transition distinguishing bisulfite modified ETS2 between (S+L^s)-rDNA and L^a-rDNA, however only in plus (GC-rich) strand. So, methylation patterns of L- and S-rDNA variants were compared within ETS2 regions I-IV, whereas (S+L^s)- and L^a-rDNAs were compared within ETS2 regions V and VI. In addition, L- and L^a –rDNAs can be compared within the partial overlap between ETS2 regions IV and V (highlighted by a pink line; Figure S8). **(b, c and e)** For each indicated pair of rDNA variants, the methylation level of each cytosine with higher specific transcriptional activity was subtracted from the methylation level of the corresponding cytosine with lower specific activity. Values for por2 leaf and root tips were averaged from por2-134 and por2-15 individuals. An asterisk indicates cytosine within motif which differs between L- and S-rDNAs and it was excluded from calculations. **(b)** Three pairs of rDNA variants (i) S and L from por2, (ii) S from por2 and por1 and (iii) L from por2 and S from por1 were compared in the regions I and II, however, only in plus (G-rich) strand. Probably increased inter-strand bias for C/G content and/or frequency of rather undermethylated CHH motifs caused large reduction of Illumina reads mapped towards minus (C- rich) strand within these regions

(Table S9). Source data are in Table S3, Sheet 1I. **(c)** S- and L- rDNAs from por2 were compared in the ETS2 regions III and IV in both strands (Table S3, Sheet 1J). **(d)** Variations in cytosine methylation differences between silent (S in por2) and active (S in por1; L in por2) rDNAs are shown for indicated motifs and tissues (statistical evaluations of results from panels B and C; Table S3, Sheet 3A). **(e)** Three pairs of rDNA variants (i) S+L^s and L^a from por2, (ii) S+L^s from por2 and S from por1 and (iii) L^a from por2 and S from por1 were compared in the ETS2 regions V and VI (Table S3, Sheet 1K). **(f)** Variations in cytosine methylation differences between silent (S+L^s in por2) and active (S in por1; L^a in por2) rDNAs are shown for indicated motifs and tissues (statistical evaluations of results from panel E. Table S3, Sheet 3B).

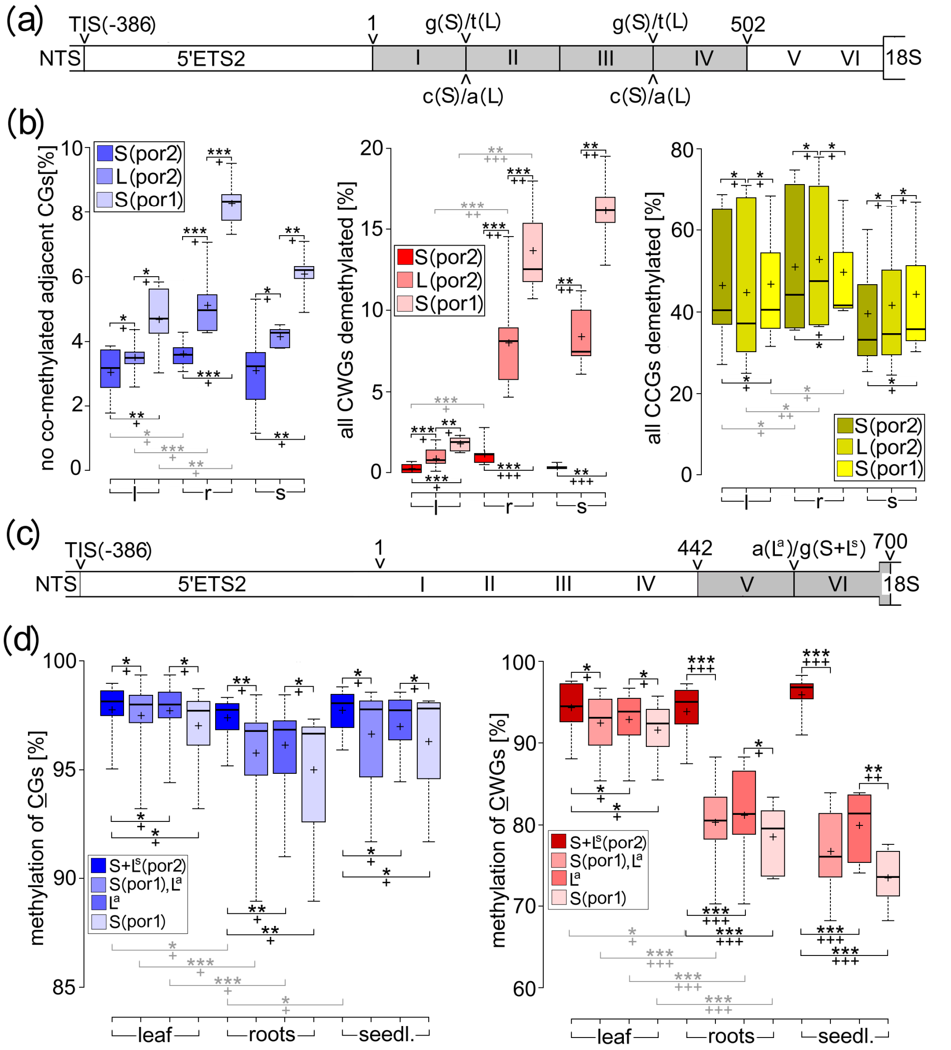

Figure S6. Methylation dynamics of individual C- motifs within individual rDNA variants – complementary data to Figure 4. (a) Locations of two g>t(c>a) substitutions which distinguish bisulfite modified L- and S- rDNA variants within ETS2 regions I-IV. **(b)** Proportions of indicated rDNAs with at least one demethylated CG from each pair of neighboring CGs or with all demethylated CWGs or CCGs. Pairwise comparisons were performed either between individual rDNA variants in leaves (l), root tips (r) and seedlings (s) (black) or between leaves and root tips for a given rDNA (gray). All comparisons are listed in

Table S3, Sheet 5B). **(c)** Location of single g>a substitution distinguishing bisulfite modified S+L^s- and L^a-rDNAs within ETS2 regions V and VI. **(d)** Methylation ranges of indicated C-motifs were compared between (i) (S+L^s)-rDNA from por2 and sum of S-rDNAs from por1 and L^a-rDNAs from por2, (ii) L^a-rDNA from por2 and S-rDNA from por1, (iii) (S+L^s)-rDNA and L^a-rDNA from por2 and (iv) (S+L^s)-rDNA from por2 and S-rDNA from por1. Selected pairwise comparisons between leaves and root tips are shown as well (gray). All comparisons are listed in Table S3, Sheet 4B. Box plots represent minimum, 1st quartile, median, 3rd quartile, maximum and mean (+). One, two and three asterisks indicate statistically unsupported ($p2$ > 0.05), moderately supported (0.01 < $p2$ < 0.05) and significantly supported ($p2$ < 0.01) differences, respectively. One, two and three crosses indicate $|D_{s-a}| < 5\%$, $5\% < |D_{s-a}| < 10\%$ and $10\% < |D_{s-a}|$, respectively (Material and Methods).

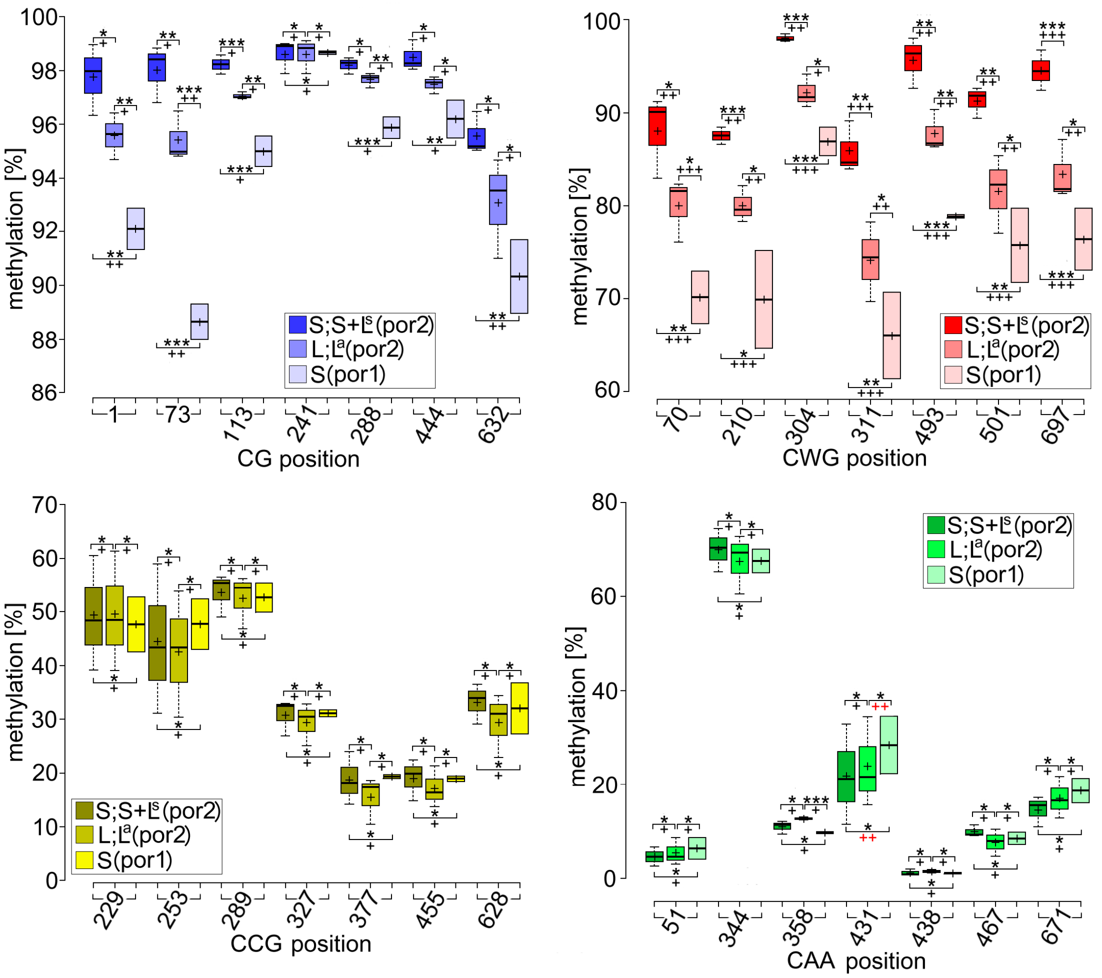

Figure S7. Comparison of methylation ranges of selected cytosines between indicated rDNA variants. Analyses of seven (six from ETS2 regions I-IV and one from ETS2 regions V-VI) representative members of each CG, CCG, CWG and CAA motif are shown. Owing to negligible methylation differences between active and silent rDNAs in leaves (Figure 4), only samples from root tips and seedlings were used for statistical analyzes. Analyses of remaining cytosines as well as analyses including all tissues or only leaves can be found in Table S3, Sheet 6A. Positions of individual motifs are according to the Figure S5a. Corresponding

analyses showing differences between all silent (S in por2) and all active (S in por1 and L in por2) can be found in Table S3, Sheet 6B. Box plots represent minimum, 1st quartile, median, 3rd quartile, maximum and mean (+). One, two and three asterisks indicate statistically unsupported ($p2 > 0.05$) moderately supported ($0.01 < p2 < 0.05$) and significantly supported ($p2 < 0.01$) differences, respectively. One, two and three crosses indicate $|D_{s\text{-}a}| < 5\%$, $5\% < |D_{s\text{-}a}| < 10\%$ and $|D_{s\text{-}a}| > 10\%$, respectively (Material and Methods). Red crosses highlight substantially lower (> 5%) methylation in leaves than in seedlings.

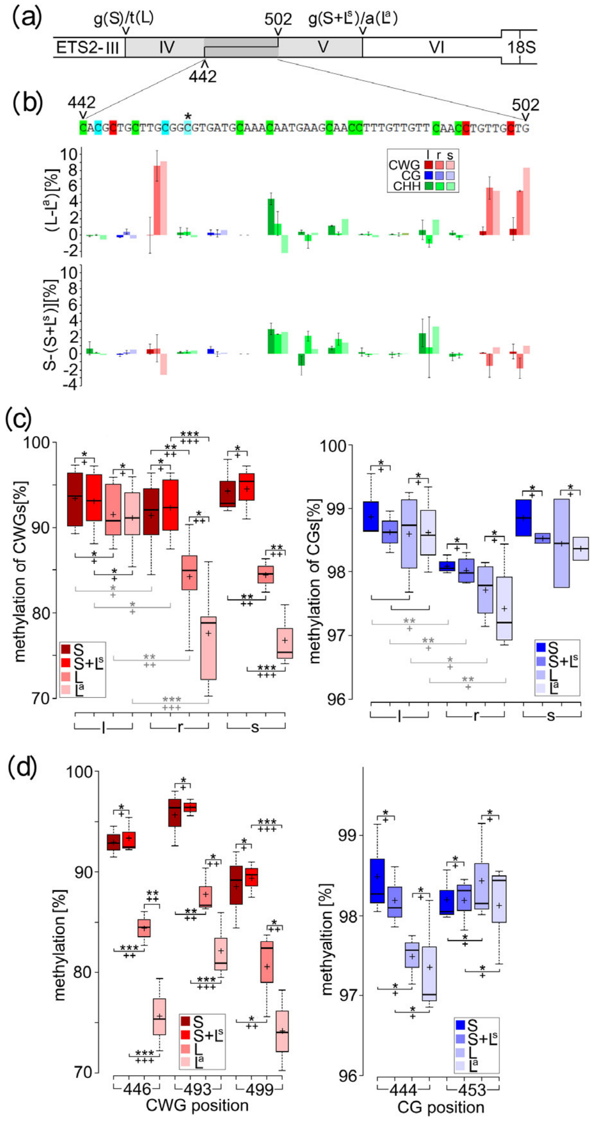

Figure S8. Cytosine methylation differences between S-, L^s- and L^a-rDNAs in por2. (a) Overlap between ETS2 region IV, where differences between S- and L- rDNAs2 were assessed based on g>t substitution, and ETS2 region V, where differences between (S+L^s)- and L^a- rDNA were assessed based on g>a substitution. **(b)** Differences in methylation of each respective cytosine between (i) L- and L^a-rDNAs and (ii) S- and (S+L^s)-rDNAs in leaves (l), root tips (r) and seedlings (s) (Table S3, Sheet 7A). **(c)** Overall methylation ranges of CWG and CG motifs were compared (i) between indicated rDNA variants in leaves (l), root tips (r) and seedlings (s) (black), and (ii) between leaves and root tips for a given rDNA variant (gray)

(Table S3, Sheet 7B). **(d)** Methylation ranges of each CWG and CG motif were compared between indicated rDNA variants. Because no differences between rDNAs were detected for leaves (panel B), only the root tips and seedlings were taken into account. Positions of individual motifs were related to panel A (Table S3, Sheet 7C). Box plots represent minimum, 1st quartile, median, 3rd quartile, maximum and mean (+). One, two and three asterisks indicate statistically unsupported (*p2* > 0.05) moderately supported (0.01 < *p2* < 0.05) and significantly supported (*p2* < 0.01) differences, respectively. One, two and three crosses indicate $|D_{s\text{-}a}| < 5\%$, $5\% < |D_{s\text{-}a}| < 10\%$ and $|D_{s\text{-}a}| > 10\%$, respectively (Material and Methods).

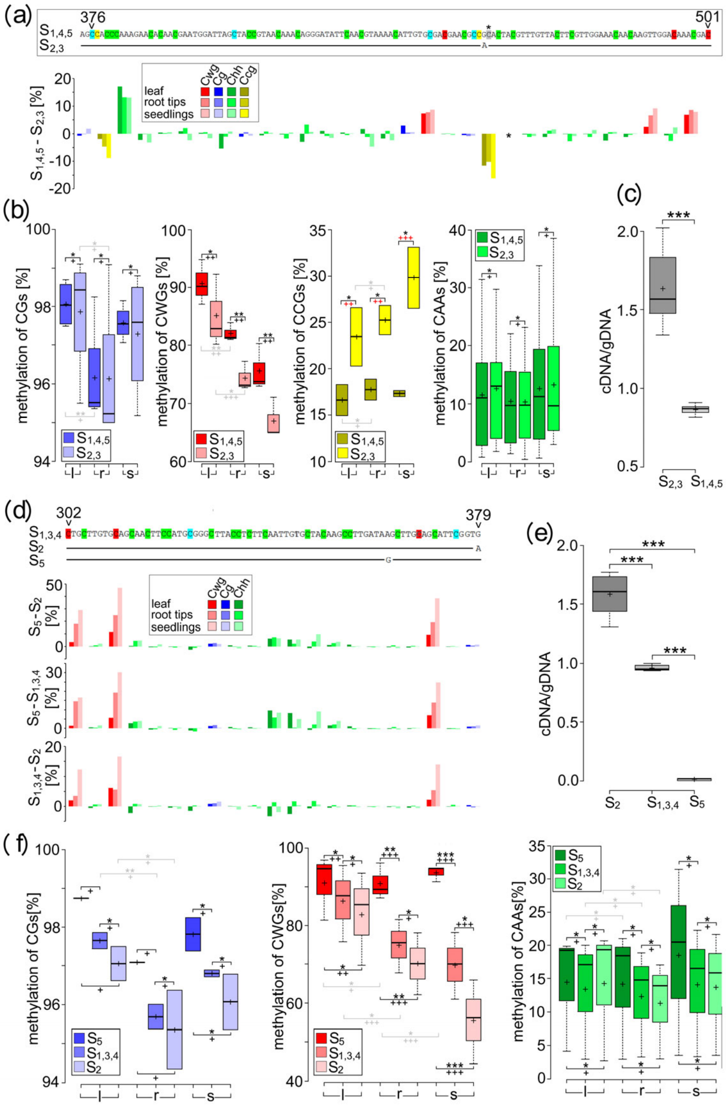

Figure S9. Comparison the methylation dynamics of individual C-motifs between individual S-rDNA variants in por1. (a) Differences in methylation of each respective cytosine between indicated S- rDNA variants distinguished by abundant G>A substitution in minus (C-rich) strand (Figure 1F). Methylation of each cytosine [%] in $S_{2,3}$–rDNAs was subtracted from the methylation of respective cytosine in $S_{1,4,5}$–rDNAs (Table A3, Sheet 8A). Numbering is related to Figure S4. An asterisk indicates cytosine within motif which differs between variants and it was excluded from evaluations**. (b)** Methylation ranges of indicated C-motifs were compared between indicated S-rDNA variants in a given tissue (black) as well

as between individual tissues for a given rDNA (gray) (Table S3, Sheet 8B). Overal methylation ranges of indicated C-motifs in all tissues together are compared between indicated S- rDNA variants in Table S3, Sheet 8C. **(c, e)** Transcriptional efficiencies (specific transcription) of indicated S- rDNA variants in por1 were expressed as the ratio between proportions of a given variant in primary transcript (cDNA) and genome (gDNA) (Table S8). **(d)** Differences in methylation of each respective cytosine between indicated S-rDNA variants distinguished by two abundant A>G and G>A transitions (Figure 1F). The methylation of each cytosine in S_2-rDNAs was subtracted from the methylation of respective cytosine either in $S_{1,3,4}$–rDNAs or in S_5-rDNA. In addition, the methylation of each cytosine in $S_{1,3,4}$ –rDNA was subtracted from the S_5 –rDNA (Table S3, Sheet 8D). **(f)** Methylation ranges of indicated C-motifs were compared between indicated S-rDNA variants in a given tissue (black) as well as between individual tissues for a given rDNA (gray) (Table S3, Sheet 8E). Overall methylation ranges of indicated C-motifs in all tissues together are compared between indicated S-rDNA variants in Table S3, sheet 8F. Box plots represent minimum, 1st quartile, median, 3rd quartile, maximum and mean (+). One, two and three asterisks indicate statistically unsupported ($p2 > 0.05$) moderately supported ($0.01 < p2 < 0.05$) and significantly supported ($p2 < 0.01$) differences, respectively. One, two and three crosses indicate $|D_{s\text{-}a}| < 5\%$, $5\% < |D_{s\text{-}a}| < 10\%$ and $|D_{s\text{-}a}| > 10\%$, respectively (Material and Methods). Pairwise comparisons were performed either between individual rDNAs in leaves (l), root tips (r) and seedlings (s) (black) or between leaves and root tips for a given rDNA (gray).

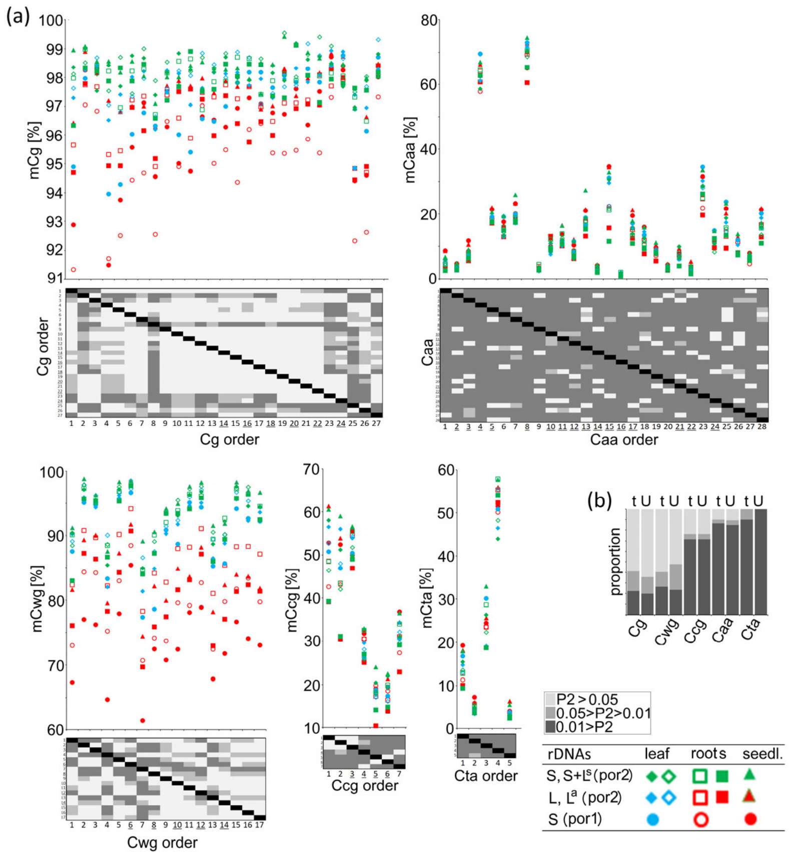

Figure S10. Cytosine methylations in ETS2 can be position-dependent, particularly for AT-rich CHH motifs. (a) Differences between methylation of individual duplicates of a given C-motif (Cg, Cwg, Ccg, Caa and Cta) were characterized by (i) methylation across the thirteen rDNA samples (charts above) and (ii) pairwise differences, statistically characterised

by t- test (below the main diagonal of the matrix), and U- test (above the main diagonal) (matrices below). The more supported the differences, the darker the shade of the given cell. Cytosines were arranged according to their order in the ETS2. Each CWG, CCG, CAA, CTA and approximately each other CG was analyzed. (b) Proportions of statistically unsupported, moderately supported and significantly supported differences are summarized as bar charts (Table S3, sheet 9A,B).
